# Novel Imprint Cytological Classification for Small Pulmonary Adenocarcinoma Using Surgical Specimens: Comparison with the 8th Lung Cancer Staging System and Histopathological Classification

**DOI:** 10.7150/jca.35027

**Published:** 2020-02-21

**Authors:** Tomoyuki Nakagiri, Tomio Nakayama, Toshiteru Tokunaga, Akemi Takenaka, Hidenori Kunoh, Hiroto Ishida, Yasuhiko Tomita, Shin-ichi Nakatsuka, Harumi Nakamura, Jiro Okami, Masahiko Higashiyama

**Affiliations:** 1 Department of General Thoracic Surgery, Osaka International Cancer Institute, Osaka, Japan; 2 Cancer Control and Statistics, Osaka International Cancer Institute Osaka, Japan; 3 Department of Cytology, Osaka International Cancer Institute, Osaka, Japan; 4 Department of Pathology, Osaka International Cancer Institute, Osaka, Japan

**Keywords:** imprint cytology, lung small adenocarcinoma, intraoperative diagnosis, cytological classification, 8^th^ IASLC classification

## Abstract

**Objectives**: Small-size lung lesions suspected of being cancer are now often being identified on computed tomography. Correspondingly, a new lung cancer staging system has been proposed by the International Association for the Study of Lung Cancer (IASLC), in which the T1 factor and adenocarcinoma are re-subclassified. Previously, we proposed an intraoperative cytological diagnosis and its classification of small-size lung adenocarcinoma, which correlated significantly with clinical malignancy, to be used for selecting the surgical strategy. In the current study, the correlation of our intraoperative cytological classification with the new 8^th^ IASLC classification was investigated.

**Materials and Methods**: A total of 139 consecutive small-size lung adenocarcinoma cases were surgically resected from 2000 to 2006 and included in this study. Intraoperative stump imprint cytology using these specimens was performed, and the cases were classified into 5 groups based on our classification. The cytological classification was compared with the IASLC classification and the WHO histopathological grading.

**Results**: According to our classification, 32 patients were in Group I, 38 in Group II, 24 in Group III, 27 in Group IV, and 18 in Group V. Compared with the IASLC classification, most of Group I was pTis or pT1mi, and most of Group II was pT1mi or pT1a (p<0.001). There was also a significant relationship between lymph node metastasis and our cytological classification (p<0.001). The histological patterns according to the WHO classification also had a significant relationship with our classification (p<0.001).

**Conclusion**: Our cytological classification correlated not only with the T classification, but also with the adenocarcinoma subclassification of the 8^th^ IASLC classification.

## Introduction

In 2015, the new Lung Cancer Stage Classification was proposed by the International Association for the Study of Lung Cancer (IASLC) according to the data from the International Union for Cancer Control (UICC) and the American Joint Committee (AJCC).^1^ In the new classification (8^th^ IASLC classification), the T1-factor has been re-subclassified, which means that the treatment for small size lung cancer has become more important than before.

In 2007, we proposed an intraoperative imprint cytological classification of small lung adenocarcinoma (Nakayama-Higashiyama's classification: N-H classification).^2^ The N-H classification was correlated with the Noguchi classification. In the present study, in order to use the classification clinically, our cytological classification was again retrospectively assessed with analyses of its relationships with the 8^th^ IASLC TNM classification system, adenocarcinoma pattern classification,^3^ and the 4^th^ WHO histological grading.^4^

## Materials and Methods

### Sample collection

**Figure 1:**
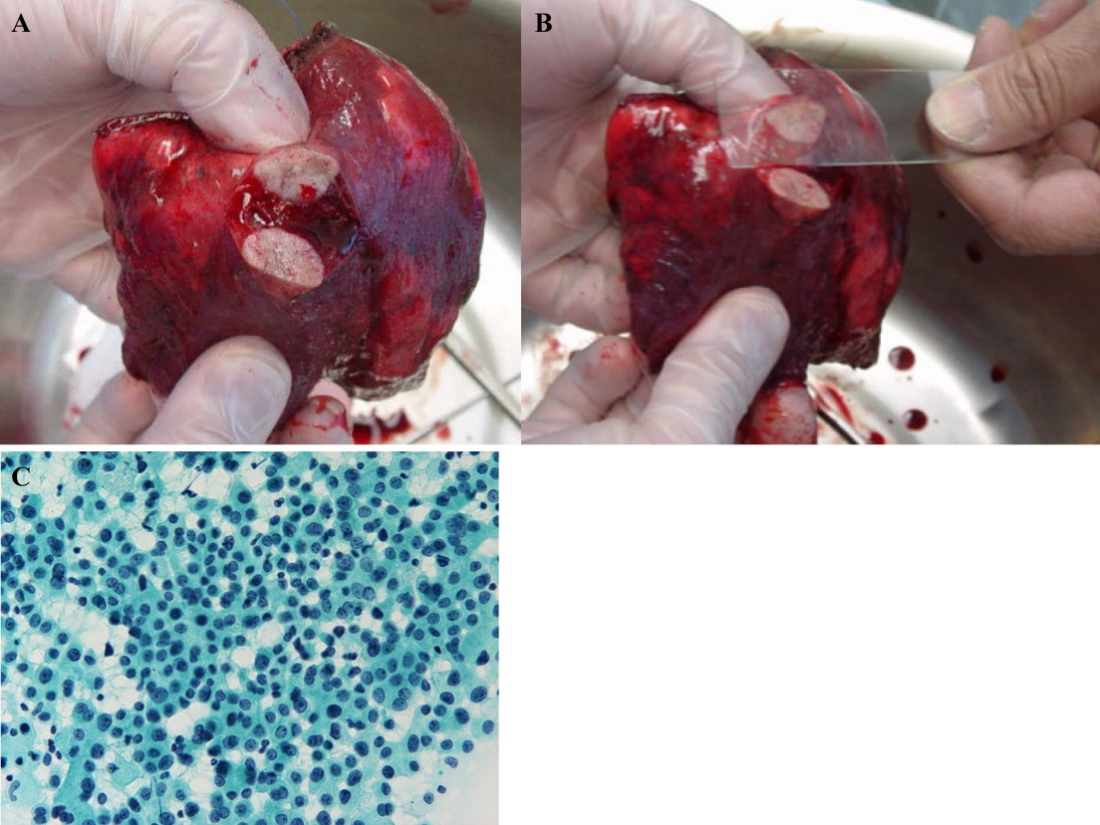
(A) The tumor was resected with a wedge or segment resection approach, then cut at the center. (B) The surface of the cut section is imprinted or smeared onto a microscope slide, and (C) then the sample is immediately fixed and observed following Papanicolaou staining.

Small size lesions in lungs with suspected lung cancer were resected by wedge resection. The tumor specimen was then cut at the center, and the stump was smeared onto a microscope slide and immediately fixed with ethanol in the operating room. Cytology specimens were examined following Papanicolaou staining (Figure 1).

### Cytological Classification (Nakayama-Higashiyama's Classification)

**Figure 2:**
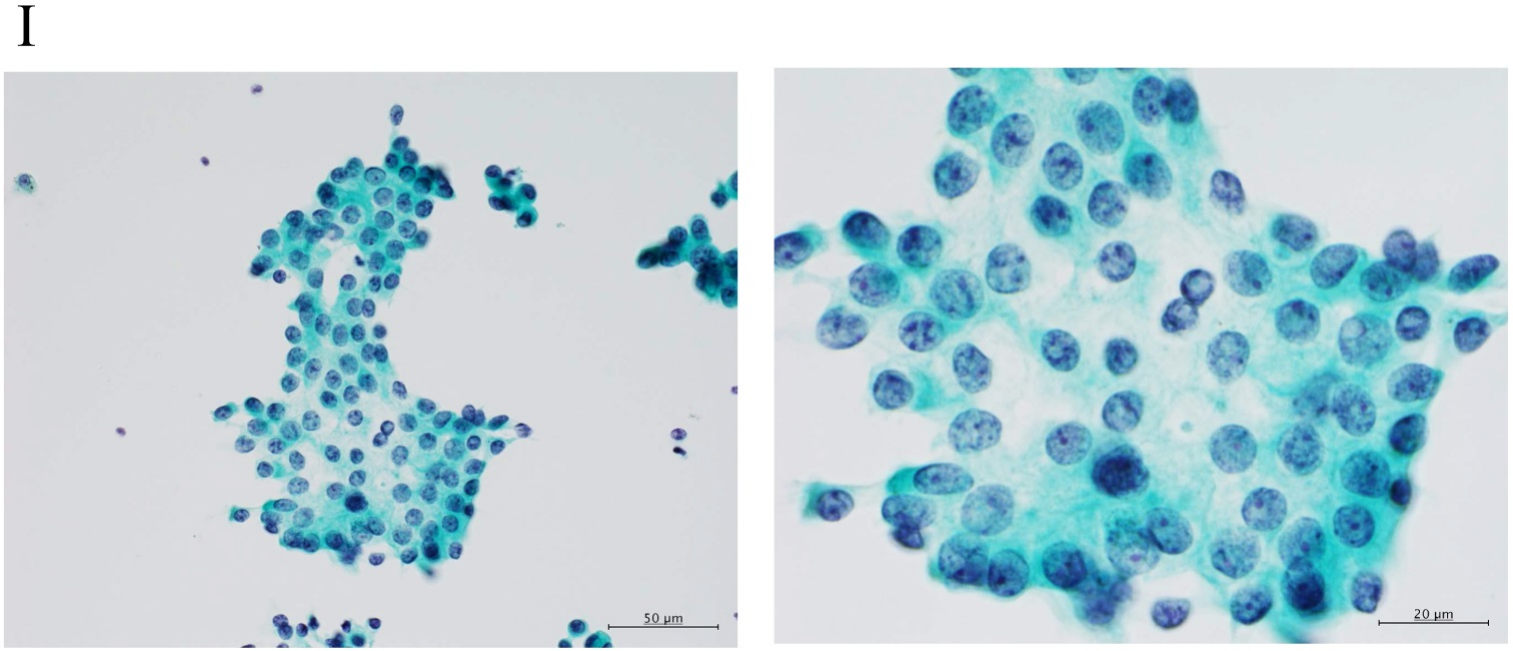

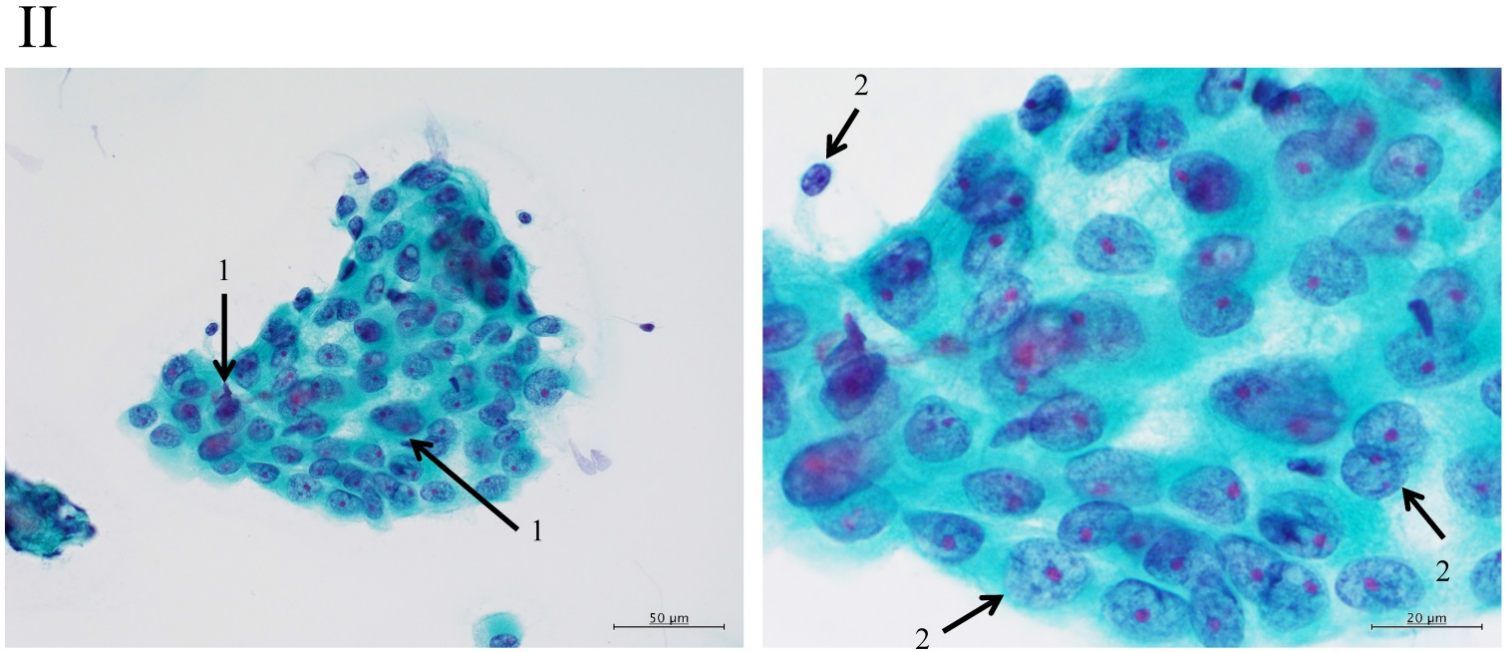

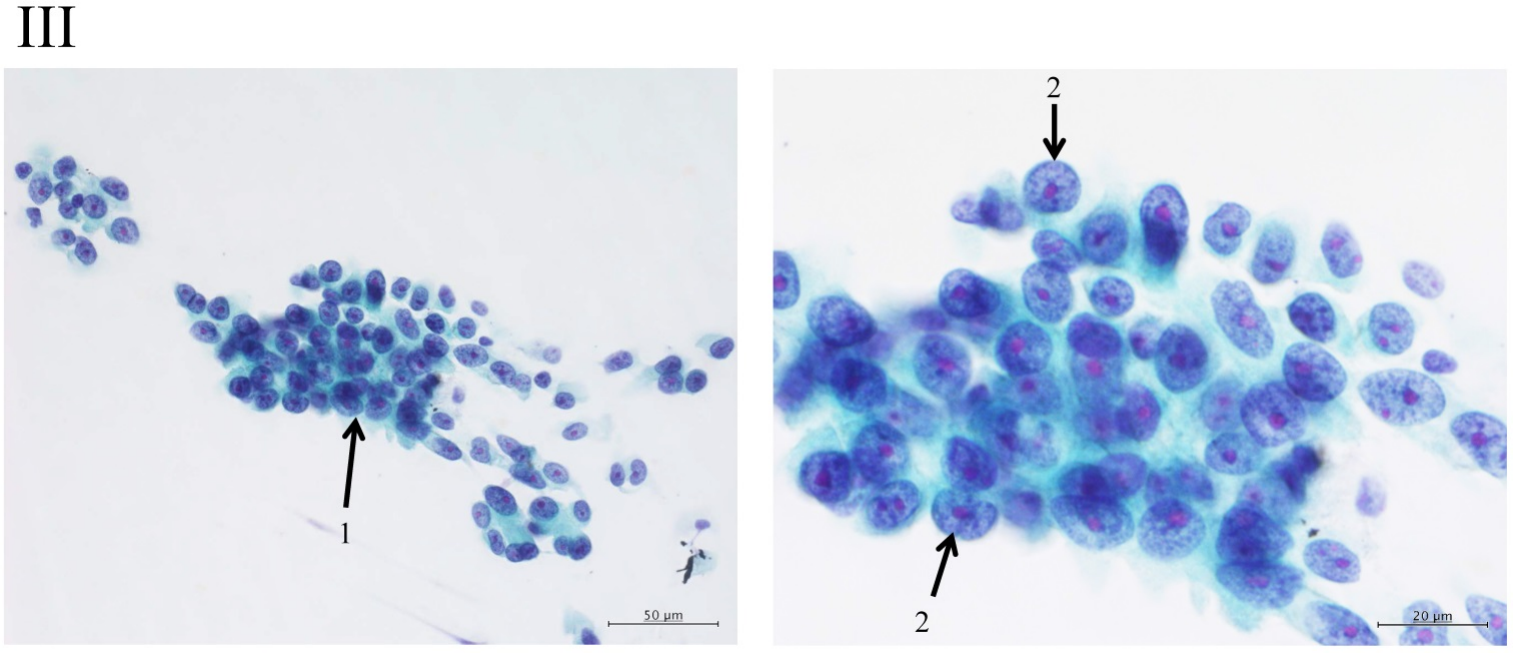

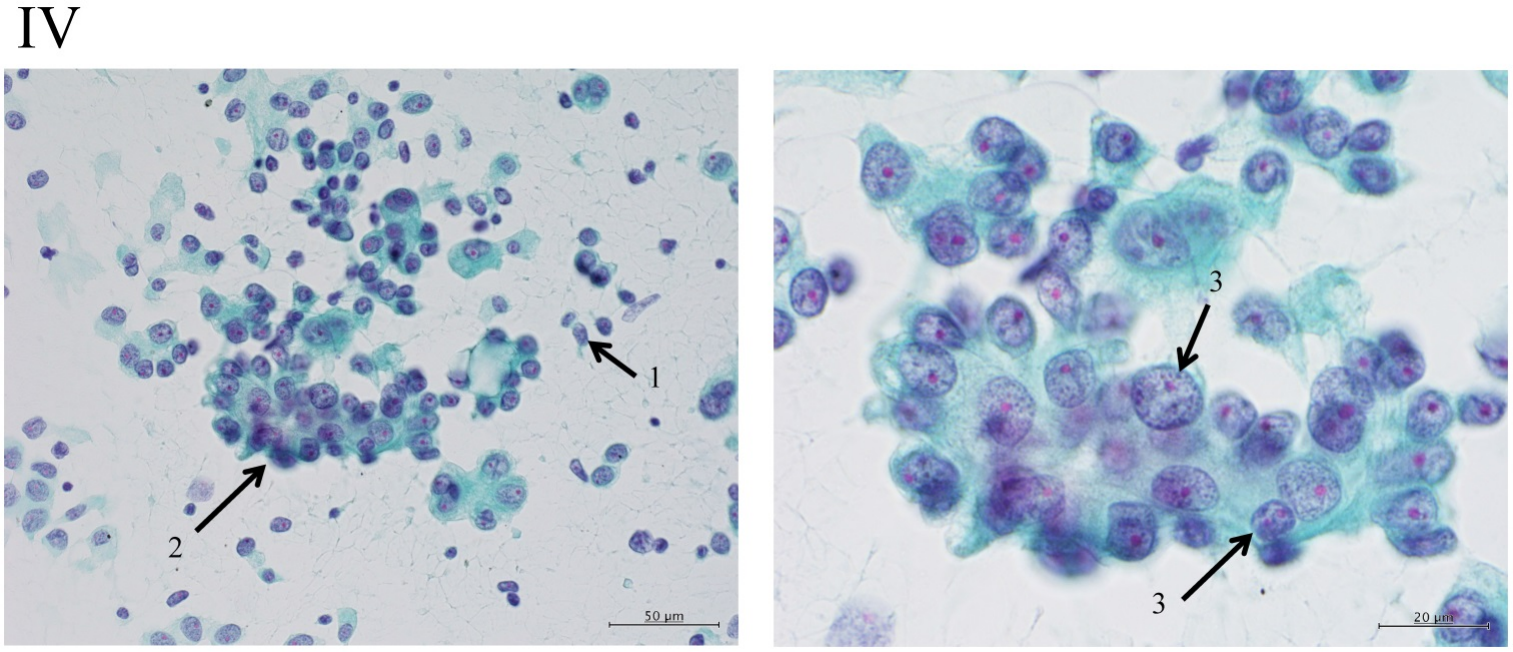

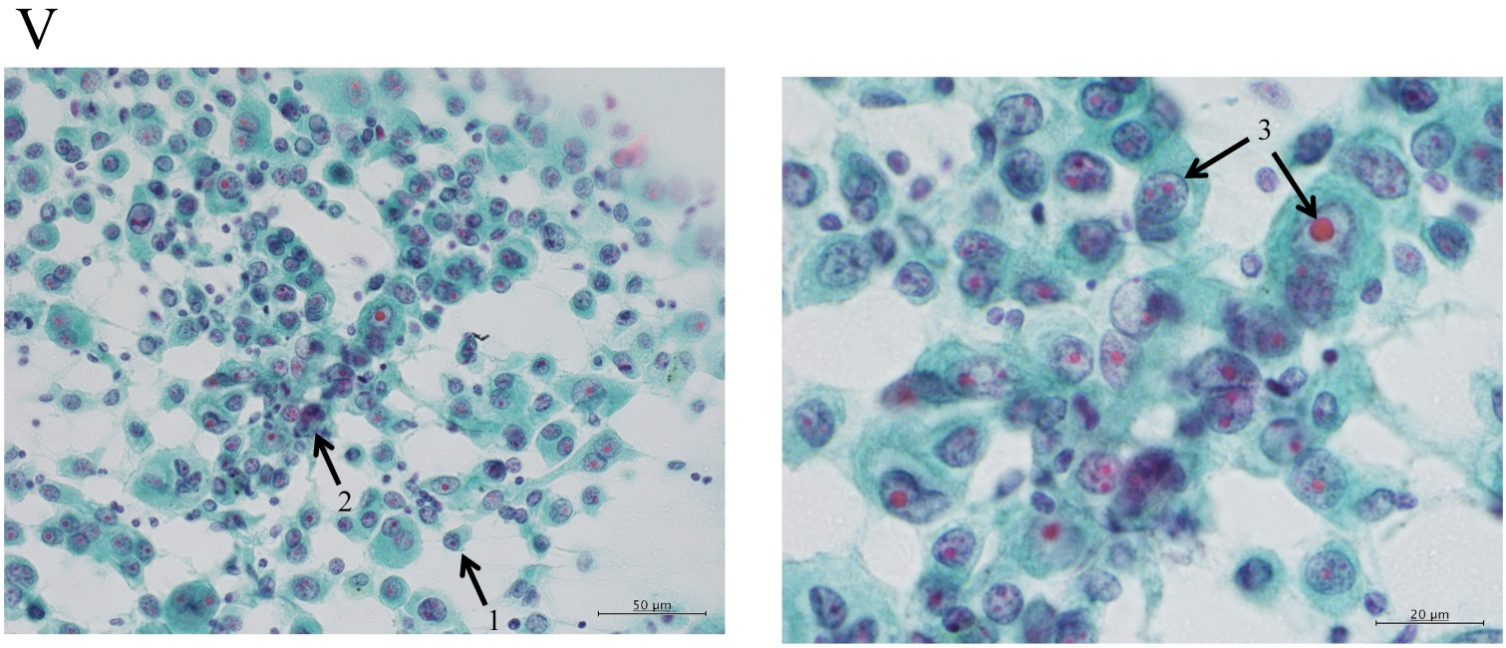
Clustering in Group I shows a sheet-like appearance without overlapping of the cells, with less nucleus inequality (I). Group II shows partially overlapped clusters (arrow 1), small nuclei, and slight anisokaryosis (arrow 2: II). In Group III, the clusters are irregular with single-cell formation (arrow 1), while papillary formation is also often seen. The chromatin pattern is fine granular with an irregular distribution (arrow 2: III). Clusters in Group IV are composed of scattered isolated cells (arrow 1) with irregular overlapping (arrow 2). The nuclei are large, and anisokaryosis is seen (arrow 3: IV). In Group V, clusters show scattered isolated cells (arrow 1) with irregular overlapping (arrow 2). The nuclei are large, and marked dyskaryosis is seen (arrow 3: V).

Adenocarcinomas smaller than 2 cm, which corresponded with T1A of the 7^th^ UICC classification, were investigated because the investigation began from 2000, excluding macroscopic mucinous adenocarcinoma cases. Cytology examiners diagnosed and classified the samples according to the N-H classification, based on cell cluster shape, cell and nucleus size, and the existence of necrosis, in a blinded manner.^2^ In the criteria, the shape of the cluster is regarded as the most important item, based on the idea of the Noguchi classification, which was classified according to the structure of the tumor. The classification criteria are shown in Table 1 and Figure 2.

### Patients

Since 1993, we have been performing intraoperative imprint cytology based on the N-H classification. In the present study, consecutive patients with small lung adenocarcinoma tumors were included. The clinical T1a and T1b N0 lung adenocarcinomas, which correspond with T1A of the 7^th^ UICC classification, according to the IASLC 8^th^ edition and that were sampled during the operations from 2000 to February 2006 were analyzed. Written, informed consent was obtained from all patients, and the study protocol was approved by the ethical review board of our institution (Approval No. 18055).

### Statistical analysis

Relationships of incidence between 2 or more groups were compared using χ^2^ contingency table analysis. Values of *p*≤0.05 were considered significant.

## Results

### Patients

A total of 139 patients/lesions were analyzed (Table 2). Whole mean tumor size was 15.3 ± 0.36 mm.

According to the 8^th^ IASLC adenocarcinoma classification and the WHO grading, 6 lesions were adenocarcinoma *in situ* (AIS, 4.3%), 41 lepidic pattern (29.5%, well differentiated [AIS + lepidic]: 33.8%), 65 papillary pattern (46.8%), 12 acinar pattern (8.6%, moderately differentiated [papillary + acinar]: 55.4%), 10 solid pattern (7.2%), and 5 mucinous pattern (3.6%, poorly differentiated [solid + mucinous]: 10.8%).

According to the new TNM classification system, lymphatic permeation was observed in 32 patients (23.0%), and vascular permeation was observed in 25 patients (18.0%). In addition, lymph node metastasis was seen in 14 patients (10.0%). For the pathological T factors, they were pTis in 6 patients (4.3%), pT1mi in 49 (35.3%), pT1a in 23 (16.5%), pT1b in 37 (26.6%), and pT2a + pT3a (PL+) in 24 (17.3%).

Using the N-H classification, there were 32 patients in Group I (23.0%), 38 in Group II (27.3%), 24 in Group III (17.3%), 27 in Group IV (19.4%), and 18 in Group V (12.9%).

### IASLC adenocarcinoma pattern classification, WHO grading, and N-H Classification

There was a significant relationship between the predominant histological patterns according to the IASLC classification and our cytological classification (p<0.0001, Table 3). In Groups I and II, adenocarcinoma *in situ* (AIS), lepidic pattern, and papillary pattern were predominant. In addition, in Groups IV and V, papillary, acinar, and solid patterns were predominant.

Comparing the WHO histological grade and our cytological classification, there was a significant relationship (p<0.0001, Table 3).

### The 8^th^ IASLC TNM classification and the N-H Classification

Compared with the pathological classification in the 8^th^ edition, there was a significant relationship with our cytological classification (p<0.001, Table 4). Most of Group I was pTis or pT1mi, and most of Group II was pT1mi or pT1a.

There was also a significant relationship between lymph node metastasis and our cytological classification (p<0.001, Table 4). In addition, lymphatic and vascular permeations also had a significant relationship with our classification (p<0.0001, both). Most of Groups I and II had no lymph node metastasis and no permeations.

## Discussion

Previously, some researchers reported postoperative imprinting cytology using resected lungs for small-size adenocarcinomas.^5^-^7^ Hoshi et al. reported that lung adenocarcinoma patients with micropapillary pattern diagnosed with intraoperative cytology had worse outcomes than the others in Stage I.^5^ Other studies reported that, diagnosed with intraoperative cytology, large cluster size, moderate or severe nuclear irregularity, many multinucleated cells, and large nuclear size were significantly associated with a poor outcome.^6^,^7^ All of these studies concluded that the cytological factors were associated with postoperative prognosis in patients with small adenocarcinoma.

We have also investigated imprinting cytology, but we focused especially on determining the criteria for malignancy potential intraoperatively and comparing it with the final pathological diagnosis. According to the previous reports, we included the aforementioned items that had significant associations with the pathological malignancy and the UICC adenocarcinoma classification in our cytological criteria. We have accumulated cases with adenocarcinoma under 2 cm since Noguchi's classification was proposed.^8^ Previously, we reported that our cytological classification was correlated with Noguchi's histological classification.^2^ However, the histological classification is already out-of-date and is not used today. In addition, the new classification of the IASLC 8^th^ edition has been published.^1^ For further clinical application, we had to verify the correlation of our cytological classification with the 8^th^ IASLC classification.

The IASLC previously proposed not only the new TNM classification, but also a detailed adenocarcinoma subclassification.^3^ In addition, the WHO proposed a histologic grading system that corresponds with the IASLC proposed adenocarcinoma classification based on the predominant histologic subtype.^4^

Our cytological classification corresponded not only with the 8^th^ IASLC TNM classification, but also its histological pattern classification. In addition, our cytological classification correlated with the WHO histological grading.

Recently, it was reported that limited pulmonary resection procedures, including a wedge resection and segmentectomy, were not inferior to lobectomy for management of certain types of peripheral small pulmonary adenocarcinomas.^9^,^10^ To perform limited pulmonary resection procedures, we must diagnose the tumor accurately.^11^

We often see small lesions in lungs with suspected cancer on computed tomography screening because of the development of diagnostic imaging technology and the distribution of preventive screening. There are some preoperative diagnostic methods, e.g. ground-glass opacity on CT and maximum standardized uptake values of positron emission tomography.^11^ However, these lesions often need to be diagnosed intraoperatively for more accurate diagnosis, with a partial lung resection including excisional biopsy for a frozen section. After the conventional intraoperative frozen diagnosis, a part of the tumor, often the center of the tumor, is lacking for the final diagnosis, although we have to describe the tumor in size, and as adenocarcinoma, the predominant type of the adenocarcinoma pattern subclassifications and so on in detail as the postoperative diagnosis according to the classification of the IASLC 8^th^ TNM system. To provide an accurate description, we must theoretically preserve the whole tumor. In this respect, our cytological method has an advantage, because the diagnosis does not need the tumor section itself, but only the surface of the section.

In conclusion, our Nakayama-Higashiyama cytological classification was found to be correlated with the histological final diagnosis, i.e. it can be used as an accurate intraoperative clinical diagnosis, because our cytological classification diagnoses not only whether the tumor is malignant, but also the malignant potential of the adenocarcinoma.

In addition, the malignant classification based on our cytological classification may help to select the procedure, because the diagnosis is performed intraoperatively. Now we apply the classification to select the operative procedure, lobectomy or sub-lobar resection, for small size adenocarcinomas. The results of such clinical application will be reported in another article (in preparation).^12^

## Limitations

This study was conducted at a single institute and had a limited number of subjects, who had all been operated on more than 10 years ago, because we have selected the surgical mode according to the result of the cytological classification since 2006. In addition, the Nakayama-Higashiyama classification is a qualitative system. To improve the classification system, a quantitative method and review of more cases are needed.

## Figures and Tables

**Table 1:** Nakayama-Higashiyama's classification of small pulmonary adenocarcinoma

	Group I	Group II	Group III	Group IV	Group V
**Cellularity**	poor	moderate	hyper	hyper	hyper
**Size of cluster**	10-30 cells	slightly large cluster	small to large cluster	single to large cluster	single to large cluster
**Shape of cluster**	sheet-like appearance	mainly sheet-like appearance, partly overlapping	irregular overlapping	scattered isolated cells to irregular overlapping	scattered isolated cells to irregular overlapping
**Size of cells**	small	small to medium	small to large	large	large
**Dyskaryosis**	none	slight	often	often	marked
**Size of nucleus**	small & uniform size	small to medium & anisokaryosis	small to large & anisokaryosis	large & anisokaryosis	large & anisokaryosis
**Chromatin pattern**	thick, fine and granular chromatin with regular distribution	thick to sparse and fine, granular chromatin	fine granular chromatin with irregular distribution	fine granular chromatin with irregular distribution	fine to coarse, granular chromatin with irregular distribution
**Distance of inter-nucleus**	slightly irregular	slightly irregular	irregular	irregular	irregular

The “Shape of cluster” is the important item in these criteria. If the cluster shapes only sheet-like appearance, the classification is into Group I or II. In addition, the two are separated according to the size of cluster. If the overlapping of cells can be seen in the cluster, the cluster is classified into Group III, IV or V. In addition, the cellularity is a matter of course “hyper”; it means the number of cells cannot be counted in the cluster. The difference between Group III and IV is mainly the cell appearance of malignancy. If the cluster has scattered isolated cells, thick overlapping of cells and/or cells with large size of nucleus, the tumor can be suspected to have high malignancy. Then the cluster is classified into Group IV or V. In addition, if the cluster has an obvious necrosis, the cluster is classified into Group V.

**Table 2:** Patients' characteristics (n=139)

Age (years, mean ± SD*)	62.3±9.8
Gender (male/female)	75 / 65
Tumor size (mm, mean ± SD)	15.3±0.36
IASLC/ATS/ERS^2^* adenocarcinoma pattern classification (AIS/L/P/A/S/M)^3^*	6 / 41 / 65 / 12 / 10 / 5
WHO grading (Well/Moderate/Poorly differentiated)	47 / 77 / 15
Nakayama-Higashiyama's classifications (Group I/II/III/IV/V)	32 / 38 / 24 / 27 / 18
pT (according to the 8^th^ IASLC classification) (Tis / T1mi / T1a / T1b)	6 / 49 / 23 / 37
Lymph node metastasis (+/-)	14 / 125
Lymphatic permeation (+/-)	32 / 107
Vascular permeation (+/-)	27 / 112

* Standard distribution. 2* IASLC/ATS/ERS: International Association for the Study of Cancer (IASLC)/ American Thoracic Society (ATS)/ European Respiratory Society (ERS) classification. 3* AI: adenocarcinoma *in situ*, L: lepidic predominant adenocarcinoma, P: papillary predominant adenocarcinoma, A: acinar predominant adenocarcinoma, S: solid predominant adenocarcinoma, M: Mucinous predominant adenocarcinoma

**Table 3:** IASLC adenocarcinoma pattern classification, WHO grading, and N-H Classification

		Nakayama-Higashiyama's cytological classification					p value
		Group I	Group II	Group III	Group IV	Group V	
		n = 32	n = 38	n = 24	n = 27	n = 18	
**IASLC adenocarcinoma pattern classification**	AIS	4	1	1	0	0	<0.0001
	Lepidic	16	14	8	3	0	
	Papillary	10	19	13	15	8	
	Acinar	0	2	1	5	4	
	Solid	0	2	0	2	6	
	Mucinous	2	0	1	2	0	
**WHO grading**	Well diff	20	10	9	3	0	<0.0001
	Moderately diff	10	26	14	20	12	
	Poorly diff	2	2	1	4	6	

**Table 4:** The 8^th^ IASLC TNM classification and N-H Classification

		Nakayama- Higashiyama's cytological classification					p value
		Group I	Group II	Group III	Group IV	Group V	
		n = 32	n = 38	n = 24	n = 27	n = 18	
**pT**	Tis	4	1	1	0	0	<0.001
	T1mi	23	17	7	2	0	
	T1a	3	7	7	4	2	
	T1b	2	5	6	17	7	
	T2/T3 (PL+)*	0	8	3	4	9	
**Lymph node metastasis**	none	32	37	23	19	14	<0.001
	N+	0	1	1	8	4	
**Lymphatic permeation**	none	31	33	18	12	13	<0.0001
	Ly+	1	5	6	15	5	
**Vascular permeation**	non	32	35	21	15	9	<0.0001
	V+	0	3	3	12	9	

*: Because of pleural invasions, the T stage was raised.
